# Supplementary material for: Comparing the Clinical and Laboratory Features of Remitting Seronegative Symmetrical Synovitis with Pitting Edema and Seronegative Rheumatoid Arthritis
Source: J Clin Med. 2021 Mar 7;10(5):1116. doi: 10.3390/jcm10051116 (PMC7962112; doi:10.3390/jcm10051116)
Supplement: Supplementary file 1 [file jcm-10-01116-s001.pdf]

Journal of Clinical Medicine

SUPPLEMENTARY MATERIAL

Comparing the clinical and laboratory features of remitting seronegative symmetrical synovitis with pitting edema and seronegative rheumatoid arthritis

Misako Higashida-Konishi [1*], Keisuke Izumi [1,2], Satoshi Hama [1], Hiroshi Takei [1], Hisaji Oshima [1], and Yutaka Okano [1]

[1] Department of Connective Tissue Diseases, National Hospital Organization Tokyo Medical Center, 1528902 Tokyo, Japan
[2] Division of Rheumatology, Department of Internal Medicine, Keio University School of Medicine, 1608582 Tokyo, Japan
* Correspondence: higashidamisako@gmail.com; Tel.: +813-3411-0111 (M.H.)

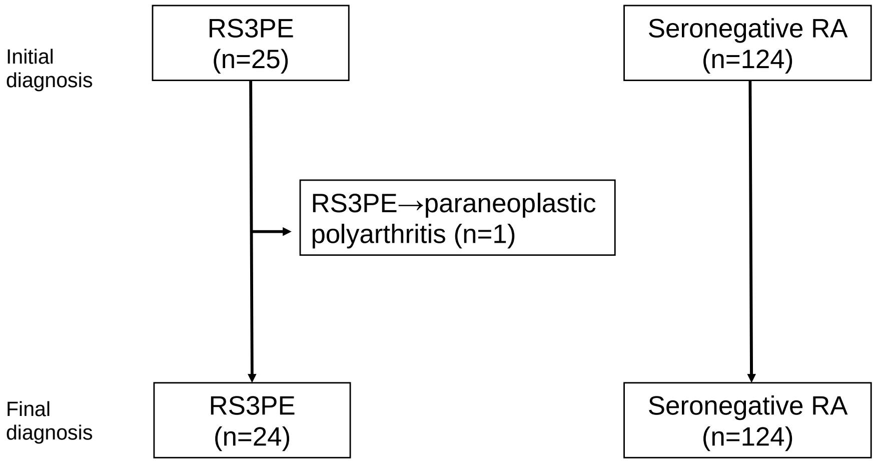

Figure S1. Flow of patient diagnosis. PMR, polymyalgia rheumatica; RA, rheumatoid arthritis; RS3PE, remitting seronegative symmetrical synovitis.

Table S1. Clinical features of the 24 patients with RS3PE at the time of diagnosis

	Patients																							
Characteristics	1	2	3	4	5	6	7	8	9	10	11	12	13	14	15	16	17	18	19	20	21	22	23	24
Age, years	53	62	68	70	73	73	76	76	77	78	78	79	80	81	83	84	84	85	87	87	88	90	91	92
Length of follow-up since the last visit, months	30	181	30	204	167	172	36	20	168	178	13	2	36	22	25	12	9	12	92	34	45	4	60	37

Sex	F	F	F	M	M	M	F	M	M	M	M	M	M	M	F	M	F	F	F	F	F	M	F	M
Smoking	N	N	N	Y	Y	N	N	Y	N	Y	N	N	Y	N	N	N	N	N	N	N	N	N	N	N
Diabetes	N	N	N	N	Y	N	Y	N	Y	N	N	N	Y	Y	N	Y	N	N	N	N	N	N	N	N
Hypertension	N	N	N	Y	N	Y	N	N	Y	N	N	N	Y	Y	Y	Y	N	Y	N	N	N	Y	N	Y
Hyperlipidemia	N	N	N	Y	Y	Y	N	N	N	N	N	N	N	N	N	Y	Y	N	N	N	N	N	N	N
Swollen or/and tender joints																								
Shoulders	N	Y	Y	N	Y	Y	N	N	Y	N	N	Y	N	Y	N	Y	N	N	N	N	N	N	N	N
Elbows	N	Y	N	N	N	N	N	N	N	N	N	Y	N	N	N	N	N	N	N	N	N	N	N	N
Wrists	Y	N	Y	Y	Y	N	Y	Y	N	N	Y	N	Y	Y	N	Y	Y	Y	Y	Y	N	Y	Y	Y
Fingers	Y	Y	Y	Y	Y	N	Y	Y	N	Y	Y	N	Y	Y	N	Y	Y	Y	Y	Y	Y	Y	N	Y
Hips	N	N	N	Y	Y	N	N	N	Y	N	N	N	N	N	N	N	N	N	N	Y	N	N	N	N
Knees	N	N	N	N	N	Y	Y	N	Y	N	N	Y	N	N	Y	N	Y	N	Y	N	Y	N	Y	N
Ankles	Y	N	Y	Y	Y	Y	Y	Y	Y	N	Y	N	Y	Y	Y	Y	Y	N	Y	Y	Y	N	Y	N
Toes	N	N	N	Y	Y	N	N	Y	N	Y	N	N	N	Y	Y	Y	N	N	N	N	N	Y	N	N
Temperature ≥38°C	N	N	N	N	Y	Y	N	N	N	N	N	N	N	N	N	N	N	N	N	N	N	N	N	N
Malaise or fatigue	N	N	N	N	N	N	N	N	N	N	Y	Y	N	N	N	N	N	N	N	N	N	N	Y	N
Weight loss	N	N	N	Y	N	N	N	N	N	N	N	Y	N	N	N	N	N	N	N	N	Y	Y	N	Y
Morning stiffness	N	Y	N	N	N	N	N	N	N	N	N	Y	N	N	N	N	N	N	N	N	N	N	N	N
Edema (both hands and feet)	Y	Y	Y	Y	Y	Y	Y	Y	Y	Y	Y	Y	Y	Y	Y	Y	Y	Y	Y	Y	Y	Y	Y	Y
Edema (only hands)	N	N	N	N	N	N	N	N	N	N	N	N	N	N	N	N	N	N	N	N	N	N	N	N
Edema (only feet)	N	N	N	N	N	N	N	N	N	N	N	N	N	N	N	N	N	N	N	N	N	N	N	N
CRP, mg/dL	1.7	9.1	3.3	7.2	0.1	13.7	5.8	9.6	14.5	6.0	5.0	10.8	15	16.9	3.8	3.1	5.7	4.4	15.9	15.0	12.6	1.5	11.7	0.7
ESR, mm/h	59	57	93	74	5	118	91	105	85	46	111	113	9.3	100	82	120	120	98	120	85	91	59	120	11
Alb, g/dL	4.2	3.9	3.7	2.7	4.6	3.9	3.5	3.8	3.3	4.0	3.5	3.2	2.9	3.1	3.6	2.7	3.6	3.5	3.0	2.7	2.9	3.9	n.d.	3.5
LDH, U/L	237	272	140	152	201	209	144	147	231	177	239	194	213	183	180	311	199	222	170	286	158	188	149	269
MMP-3, ng/mL	46	n.d.	85	n.d.	n.d.	n.d.	681	435	n.d.	n.d.	398	269	2848	235	320	319	1313	101	605	414	3316	359	n.d.	n.d.
Hb, g/dL	14	12	11	12	14	12	11	13	9	12	10	11	7	11	11	8	9	10	10	10	10	12	10	10
Malignancy (within 2 years before and after the diagnosis of RS3PE)	N	N	N	N	N	N	N	N	N	Y	Y	Y	Y	Y	N	N	N	N	N	Y	N	N	N	N
Patients fulfilling the classification criteria for RA [11, 12]	Y	N	N	Y	Y	N	N	N	N	Y	N	N	N	Y	N	N	N	N	N	N	Y	N	N	Y
Patients fulfilling the classification criteria for PMR [10]	N	Y	N	N	N	N	N	N	N	N	N	Y	N	N	N	N	N	N	N	N	N	N	N	N

Alb, albumin; CRP, C-reactive protein; ESR, erythrocyte sedimentation rate; F, Female; Hb, hemoglobin; LDH, lactate dehydrogenase; M, Male; MMP-3, matrix metalloproteinase 3; N, No; n.d., no data; PMR, polymyalgia rheumatica; RA, rheumatoid arthritis; RS3PE, remitting seronegative symmetrical synovitis with pitting edema; SD, standard deviation; Y, yes

Table S2. Baseline characteristics at diagnosis of RS3PE and seronegative RA patients, excluding patients fulfilling the classification criteria for PMR.

**Characteristics**	**RS3PE (n＝22)**		**Seronegative RA (n＝107)**		**p**
Age, median (IQR), years	80.5 (75.3–87.0)		69.0 (60.0–79.0)		<0.001
Length of follow–up, median (IQR), months	31.5 (15.5–95.9)		61.9 (30.5–92.3)		0.09
Male sex, n (%)	12 (54.6)		36 (33.6)		0.09
Smoking, n (%)	5 (22.7)		18 (16.2)		0.54
Diabetes, n (%)	6 (27.3)		12 (11.2)		0.08
Hypertension, n (%)	12 (54.6)		35 (32.7)		0.09
Hyperlipidemia, n (%)	5 (22.7)		31 (29.0)		0.61
Swollen or/and tender joints, n (%)					
Shoulders	6 (27.3)		50 (45.7)		0.10
Elbows	0 (0.0)		39 (36.5)		<0.001
Wrists	17 (77.3)		84 (78.5)		1.00
Fingers	18 (81.8)		103 (96.3)		0.029
Hips	3 (13.6)		10 (9.4)		0.46
Knees	8 (36.4)		50 (46.7)		0.48
Ankles	18 (81.8)		56 (52.3)		0.017
Toes	8 (36.4)		31 (29.0)		0.61
Patients with swollen large joints, n (%)	16 (72.7)		53 (49.5)		0.06
Patients with swollen small joints, n (%)	20 (90.9)		107 (100.0)		0.028
Number of swollen large joints, median (IQR), n	2.0 (0.0–3.0)		0.0 (0.0–2.0)		0.10
Number of swollen small joints, median (IQR), n	3.5 (1.8–14.5)		9.0 (5.0–15.0)		0.038
28 swollen joints, median (IQR), n	4.5 (1.0–11.3)		8.0 (5.0–14.0)		0.040
28 tender joints, median (IQR), n	6.5 (4.8–12.3)		10.0 (7.0–15.0)		0.052
Patients with erosion, n (%)	0 (0.0)		38 (35.5)		<0.001
Systemic signs and symptoms, n (%)					
Temperature ≥38℃	2 (9.1)		7 (6.6)		0.65
Malaise or fatigue	2 (9.1)		5 (4.7)		0.34
Weight loss	4 (18.2)		9 (8.4)		0.23
Morning stiffness (lasting at least 1 hour)	0 (0.0)		18 (16.8)		0.52
Edema (both hands and feet)	22 (100)		0 (0.0)		<0.001
Edema (only hands)	0 (0.0)		1 (1.0)		1.00
Edema (only feet)	0 (0.0)		18 (16.8)		0.041
CRP, median (IQR), mg/dL	6.6 (3.8–13.8)		2.7 (0.6–6.2)		0.001
ESR, median (IQR), mm/h					
Men+Women	91.0 (59.0–111.5)		55.5 (31.5–90.0)	(n=106)*	0.013

Men	79.5 (19.8–109.5)		58.0 (29.0–90.0)	(n=35) *	0.63
Women	92.0 (84.3–114.8)		54.0 (32.0–88.0)	(n=71) *	0.003
Alb, median (IQR), g/dL	3.5 (3–3.7)	(n=21) *	3.9 (3.4–4.2)	(n=87) *	0.008
LDH, median (IQR), U/L	196.5 (156.5–232.5)		176.0(157.0–195.0)		0.044
MMP-3, median (IQR), ng/mL					
Men+Women	397.6(234.7–681.3)	(n=15) *	173.0(78.1–424.6)	(n=99) *	0.013
Men	378.5(297.8–1038.7)	(n=6) *	212.0(114.6–424.6)	(n=35) *	0.039
Women	414.1(92.8–997.2)	(n=9) *	157.0(47.8–449.0)	(n=64) *	0.10
Hb, mean±SD, g/dL					
Men+Women	10.7±1.8		11.8±1.8		0.005
Men	10.8±2.1		12.1±1.7		0.10
Women	10.5±1.5		11.7±1.8		0.041
Malignancy (within 2 years before and after the diagnosis of RS3PE or seronegative RA), n (%)	5 (22.7)		8 (7.5)		0.046
Patients fulfilling the classification criteria for RA [11, 12], n (%)	7 (31.8)		107 (100.0)		<0.001

Alb, albumin; CRP, C-reactive protein; ESR, erythrocyte sedimentation rate; Hb, hemoglobin; IQR, interquartile range; LDH, lactate dehydrogenase; MMP-3, matrix metalloproteinase 3; PMR, polymyalgia rheumatica; RA, rheumatoid arthritis; RS3PE, remitting seronegative symmetrical synovitis with pitting edema; SD, standard deviation. * In the case of missing data, the number of patient with available data was specified.

Table S3. Baseline characteristics in patients with RS3PE at diagnosis.

**Characteristics**	**With malignancy (n=6)**		**Without malignancy (n=18)**		**P value**
Age, median (IQR), years	79.5 (78.0–82.5)		80.0 (72.3–87.3)		0.76
Length of follow–up, median (IQR), months	25.9 (7.9–95.9)		31.5 (15.2–164.7)		0.53
Male sex, n (%)	5 (83.3)		8 (44.4)		0.16
Smoking, n (%)	2 (33.3)		3 (16.7)		0.57
Diabetes, n (%)	2 (33.3)		4 (22.2)		0.62
Hypertension, n (%)	2 (33.3)		10 (55.6)		0.64
Hyperlipidemia, n (%)	0 (0.0)		5 (27.8)		0.28
Swollen or/and tender joints, n (%)					
Shoulders	2 (33.3)		6 (33.3)		1.00
Elbows	1 (16.7)		1 (5.6)		0.45
Wrists	4 (66.7)		13 (72.2)		1.00
Fingers	5 (83.3)		14 (77.8)		1.00
Hips	1 (16.7)		3 (16.7)		1.00

Knees	1 (16.7)		8 (44.4)		0.22
Ankles	4 (66.7)		14 (77.8)		0.59
Toes	2 (33.3)		6 (33.3)		1.00
Patients with swollen large joints, n (%)	5 (83.3)		12 (66.7)		0.63
Patients with swollen small joints, n (%)	5 (83.3)		16 (88.9)		0.78
Number of swollen large joints, median (IQR), n	2.5 (0.75–4)		2.0 (0–2.0)		0.27
Number of swollen small joints, median (IQR), n	8.0 (1.5–26.8)		3.0 (1.8–11.0)		0.44
28 swollen joints, n	3.5 (0.8–14.5)		4.0 (1.8–10.3)		0.84
28 tender joints, n	6.0 (3.5–14.5)		7.5 (4.8–12.3)		0.90
Patients with erosion, n (%)	0 (0.0)		0 (0.0)		
Systemic signs and symptoms, n (%)					
Temperature ≥38℃	0 (0.0)		2 (11.1)		1.00
Malaise or fatigue	2 (33.3)		1 (5.6)		0.14
Weight loss	1 (16.7)		4 (22.2)		0.77
Morning stiffness (lasting at least 1 hour)	1 (16.7)		1 (5.6)		0.15
Edema (both hands and feet)	6 (100.0)		18 (100.0)		
Edema (only hands)	0 (0.0)		0 (0.0)		
Edema (only feet)	0 (0.0)		0 (0.0)		
CRP, median (IQR), mg/dL	12.9 (5.7–16.2)		6.5 (2.9–12.9)		0.10
ESR, median (IQR), mm/h					
Men+Women	105.5 (36.8–114.8)		88.0 (59.0–108.3)		0.69
Men	100.0 (27.7–115.5)		79.5 (23.0–114.8)		0.83
Women	113.0		91.0 (76.3–103.5)		0.34
Alb, median (IQR), g/dL	3.2 (3.0–3.6)		3.5 (3.1–3.8)		0.38
LDH, median (IQR), U/L	188.5 (175.3–219.5)		200.0 (151.3–245.0)		0.63
MMP-3, median (IQR), ng/mL					
Men+Women	397.6 (251.9–1726.8)	(n=5) *	359.4 (100.6–681.3)	(n=11) *	0.78
Men	333.4 (243.3–2235.8)	(n=4) *	333.4 (318.8–435.4)	(n=3) *	0.72
Women	605.0	(n=1) *	367.1 (88.8–1155.1)	(n=8) *	0.70
Hb, mean±SD, g/dL					
Men+Women	10.2±1.7		10.9±1.8		0.33
Men	10.3±1.9		11.2±2.2		0.24
Women	9.5		10.7±1.5		0.26
Patients fulfilling the classification criteria for RA [11, 12], n (%)	2 (33.3)		5 (27.8)		1.00
Patients fulfilling the classification criteria for PMR [10], n (%)	1 (16.7)		1 (5.6)		0.45

Alb, albumin; CRP, C-reactive protein; ESR, erythrocyte sedimentation rate; Hb, hemoglobin; IQR, interquartile range; LDH, lactate dehydrogenase; MMP-3, matrix metalloproteinase 3; PMR, polymyalgia rheumatica; RA, rheumatoid arthritis; RS3PE, remitting seronegative symmetrical synovitis with pitting edema; SD, standard deviation

*In the case of missing data, the number of patient with available data was specified.

Table S4. Baseline characteristics in patients with seronegative RA at diagnosis

**Characteristics**	**With malignancy (n=8)**	**Without malignancy (n=116)**	**P value**
Age, median (IQR), years	74.5 (64.8–82.8)	68.0 (58.0–77.8)	0.18
Length of follow–up, median (IQR), months	58.3 (31.2–85.2)	62.9 (30.7–85.2)	0.76
Male sex, n (%)	5 (62.5)	41 (35.3)	0.15
Smoking, n (%)	3 (37.5)	20 (17.2)	0.16
Diabetes, n (%)	2 (25.0)	12 (10.3)	0.22
Hypertension, n (%)	3 (37.5)	38 (32.8)	1.00
Hyperlipidemia, n (%)	4 (50.0)	29 (25.0)	0.21
Swollen or/and tender joints, n (%)			
Shoulders	3 (37.5)	64 (55.2)	0.47
Elbows	4 (50.0)	49 (42.2)	0.72
Wrists	7 (87.5)	93 (80.2)	1.00
Fingers	8 (100.0)	112 (96.6)	1.00
Hips	1 (12.5)	12 (10.3)	1.00
Knees	4 (50.0)	55 (47.4)	1.00
Ankles	4 (50.0)	61 (52.6)	1.00
Toes	2 (25.0)	33 (28.5)	1.00
Patients with swollen large joints, n (%)	1 (12.5)	63 (54.3)	0.029
Patients with swollen small joints, n (%)	8 (100.0)	116 (100.0)	1.00
Number of swollen large joints, median (IQR), n	0.0 (0.0–0.0)	1.0 (0.0–2.0)	0.027
Number of swollen small joints, median (IQR), n	14.5 (7.3–17.5)	9.0 (5.0–13.0)	0.12
28 swollen joints, median (IQR), n	15.0 (5.5–18.3)	8.0 (4.3–13.8)	0.18
28 tender joints, median (IQR), n	13.0 (6.3–19.8)	11.0 (8.0–15.0)	0.70
Patients with erosion, n (%)	5 (62.5)	34 (29.3)	0.11
Systemic signs and symptoms, n (%)			
Temperature ≥38℃	0 (0.0)	7 (6.0)	1.00
Malaise or fatigue	0 (0.0)	8 (6.9)	1.00
Weight loss	0 (0.0)	12 (10.3)	1.00
Morning stiffness (lasting at least 1 hour)	3 (37.5)	28 (24.1)	0.41
Edema (both hands and feet)	0 (0.0)	0 (0.0)	
Edema (only hands)	0 (0.0)	1 (0.9)	1.00
Edema (only feet)	0 (0.0)	19 (16.4)	0.61
CRP, median (IQR), mg/dL	4.9 (0.3–7.9)	2.7 (0.7–6.6)	0.89
ESR, median (IQR), mm/h			

Men+Women	26.0 (19.0–90.0)		56.0 (32.3–89.8)		0.20
Men	68.0 (29.5–110.3)		57.0 (31.0–90.0)		0.78
Women	19.0 (12.0–26.0)		55.0 (33.0–88.0)		0.020
Alb, median (IQR), g/dL	3.7 (3.5–4.2)		3.7 (3.5–4.2)	(n=92) *	0.86
LDH, median (IQR), U/L	170.5 (145.8–208.0)		176.0 (155.5–195.0)		0.57
MMP-3, median (IQR), ng/mL					
Men+Women	83.8 (36.5–201.5)		173.0 (83.7–424.6)	(n=107) *	0.07
Men	146.0 (83.8–276)		211.5 (115.0–425.8)	(n=40) *	0.46
Women	35.3 (25.7–40.1)		152.4 (48.8–401.1)	(n=67) *	0.026
Hb, mean±SD, g/dL					
Men+Women	11.5±2.1		11.9±1.8		0.51
Men	10.2±1.0		12.5±1.6		0.004
Women	13.7±1.3		11.6±1.8		0.045
Patients fulfilling the classification criteria for PMR [10], n (%)	0 (0.0)		17 (14.6)		0.60

Alb, albumin; CRP, C-reactive protein; ESR, erythrocyte sedimentation rate; Hb, hemoglobin; IQR, interquartile range; LDH, lactate dehydrogenase; MMP-3, matrix metalloproteinase 3; PMR, polymyalgia rheumatica; RA, rheumatoid arthritis; SD, standard deviation. *In the case of missing data, the number of patient with available data was specified.

© 2020 by the authors. Submitted for possible open access publication under the terms and conditions of the Creative Commons Attribution (CC BY) license (http://creativecommons.org/licenses/by/4.0/).
